# Investigating the unaccounted ones: insights on age-dependent reproductive loss in a viviparous fly

**DOI:** 10.3389/fevo.2023.1057474

**Published:** 2023-06-09

**Authors:** Sinead English, Antoine M.G. Barreaux, Robert Leyland, Jennifer S. Lord, John W. Hargrove, Glyn A. Vale, Lee R. Haines

**Affiliations:** 1 School of Biological Sciences, University of Bristol, Bristol, United Kingdom; 2 Intertryp, Université Montpellier, CIRAD, IRD, Montpellier, France; 3 Animal Health Theme, ICIPE, Nairobi, Kenya; 4 Liverpool School of Tropical Medicine, Liverpool, United Kingdom; 5 South African Centre for Epidemiological Modelling and Analysis, Stellenbosch University, Stellenbosch, South Africa; 6 National Resources Institute, University of Greenwich, Chatham, United Kingdom; 7 Department of Biological Sciences, University of Notre Dame, Notre Dame, IN, United States

**Keywords:** tsetse, pregnancy loss, prenatal mortality, reproductive senescence, offspring quality

## Abstract

Most empirical and theoretical studies on reproductive senescence focus on observable attributes of offspring produced, such as size or postnatal survival. While harder to study, an important outcome of reproduction for a breeding individual is whether a viable offspring is produced at all. While prenatal mortality can sometimes be directly observed, this can also be indicated through an increase in the interval between offspring production. Both direct reproductive loss and presumed losses have been found to increase in older females across several species. Here, we study such reproductive loss (or “abortion”) in tsetse, a viviparous and relatively long-lived fly with high maternal allocation. We consider how age-dependent patterns of abortion depend on the developmental stage of offspring and find that, as per previous laboratory studies, older females have higher rates of abortion at the late-larval stage, while egg-stage abortions are high both for very young and older females. We track the reproductive output of individual females and find little evidence that experiencing an abortion is an adaptive strategy to improve future reproductive outcomes. After an abortion, females do not generally take less time to produce their next offspring, these offspring are not larger, and there is no sex bias towards females, the sex with presumed higher fitness returns (being slightly larger and longer-lived than males, and with high insemination rates). Abortion rates are higher for breeding females experiencing stress, measured as nutritional deprivation, which echoes previous work in tsetse and other viviparous species, i.e., humans and baboons. We discuss our results in the context of studies on reproductive loss across taxa and argue that this is an important yet often overlooked reproductive trait which can vary with maternal age and can also depend on environmental stressors.

## 1 Introduction

Older breeders tend to have reduced reproductive performance, producing smaller or poorer quality offspring with shorter lifespans (Monaghan et al., 2008, 2020; Ivimey-Cook and Moorad, 2020). There is extensive empirical evidence for such reproductive senescence across taxa, both in the wild (Berman et al., 2009; Nussey et al., 2009; Sharp and Clutton-Brock, 2010; Lemaître and Gaillard, 2017) and laboratory (Miller et al., 2014; de Boer et al., 2018; Lord et al., 2021). Many studies also find that younger breeders have lower reproductive output (e.g., Zedrosser et al., 2009; Lord et al., 2021). This results in a bell-shaped (i.e., inverse U-shaped) pattern of offspring size or quality with parental age. Our focus here is on species where females carry the main costs of reproduction; accordingly, hereafter, we consider specifically the effect of maternal age.

Several theoretical models have investigated why bell-shaped, age-dependent allocation might be optimal. In terms of the later-life decline, there may be antagonistic links between traits that favour early survival or reproduction even if these come at a cost of later-life output (Kirkwood, 1977), particularly as the strength of selection declines with age. Moreover, given that reproduction can result in physiological damage, older females may strategically reduce investment in reproduction to reduce damage-induced mortality (McNamara et al., 2009). The bell-shaped curve may be associated with extrinsic mortality, such as predation (Cichoń, 2001), or age-specific trends in energy intake or the physiological costs of reproduction (Barreaux et al., 2022).

While reproductive output tends to be measured in terms of offspring quality (size or physiological condition), one might also predict age-dependent effects on prenatal mortality or early reproductive failures (termed “spontaneous abortion” or “abortion” hereafter). There are several reasons for spontaneous abortion to be higher at certain ages, in particular for very young or old mothers, based on theories about reproductive senescence (e.g., Kirkwood, 1977; McNamara et al., 2009). First, where there are trade-offs between investing in offspring production versus self-maintenance, breeding females may favour self-preservation when they are younger, and have a longer potential reproductive lifespan ahead, even if this comes at a risk of higher offspring mortality (the “adaptive” hypothesis). Second, the risk of spontaneous abortion may reflect physiological constraints, for example on the amount of energy available to invest in offspring, which could be lower in young, inexperienced mothers (the “constraint” hypothesis). Related to this, as females get older, they could accumulate physiological damage, from cellular processes (such as damage associated with reactive oxygen species) through to organ or other anatomical deterioration, and thus older females could be in compromised physiological condition to produce viable offspring. Both adaptive and constraint hypotheses have implications for whether abortions happen early or later during the pregnancy period, i.e., depending on how much has already been invested; and for whether abortions are more likely to occur for a particular sex, depending on sex differences in fitness returns, or in the energetic requirements of male or female offspring.

Comparative data on age-specific abortions across animals are rare, owing to difficulties in observing pre-natal mortality (Graham, 1979). There is considerable evidence across human populations that rates of pre-natal mortality, termed miscarriage, increase with maternal age at conception (Forbes, 1997; Nybo Andersen et al., 2000; Brosens et al., 2022; Zhang et al., 2022), are slightly higher in young mothers (Brosens et al., 2022), and miscarriages are more prevalent in the first trimester of pregnancy (Ammon Avalos et al., 2012; Brosens et al., 2022). Rather than focusing on age-specific effects, most studies in non-human species find that spontaneous abortions increase with maternal stress (Young et al., 2006; Cant et al., 2010), or occur when pregnant females are exposed to an unfamiliar male (Bruce, 1960; Roberts et al., 2012). A recent study in wild baboons has shown, however, that both age and ecological factors affect pregnancy failures (Fogel et al., 2023): younger and older females have higher rates of foetal loss, as do females experiencing heat stress or poor habitats. Studies in dolphins and macaques show that interbirth intervals increase with maternal age (Paul et al., 1993; Karniski et al., 2018), which could be indicative of an increased risk of spontaneous abortion. As these species also exhibit postnatal care, however, such intervals may instead be due to more prolonged care and lower conception rates among older females.

Here, we investigate age-dependent patterns of abortion in live-bearing tsetse (*Glossina* spp.). Female tsetse give birth to a single larva, which, after developing through three instar stages *in utero*, is almost the same weight as the mother (Benoit et al., 2015; Haines et al., 2020). Furthermore, after birth, the female immediately starts the next reproductive cycle using stored sperm and is thus in an almost continuous state of pregnancy (Denlinger and Ma, 1974). Because of this unique life history, female tsetse are excellent models for studying reproductive senescence due to their high maternal investment, iteroparous reproduction and relatively long lifespans, living up to 8 months in the field (Carpenter, 1913). Moreover, as tsetse females do not provide postnatal care, patterns of interbirth interval reflect *in utero* constraints or strategies and are not confounded by subsequent investment.

One might expect abortion rates to increase with age in tsetse due to reproductive senescence, yet mixed evidence is reported in the literature. Laboratory studies indicate that the probability of spontaneous larval abortion increases with female age (Lord et al., 2021). Estimated abortion rates in field populations are low, however, and do not increase with age (Hargrove, 1999). On the contrary, if empty uteri can be taken as evidence for a recent abortion, it seems that in the field young females have relatively high abortion rates (Hargrove, 1999), and that the rates are not high in older flies (Hargrove, 2023). Physiological and environmental stress are stronger predictors of overall abortion than age in tsetse, in both field and laboratory studies. Abortion rates are elevated for females in low nutritional condition (Mellanby, 1937; Saunders, 1972; Lord et al., 2021) and for tsetse exposed to insecticides (Riordan, 1986). While abortions are infrequent in wild tsetse, they do increase during hotter months of the year (Hargrove, 1999, 2023), when flies exhibit increased mortality levels (Hargrove, 2001) and are likely experiencing more physiological stress, due for example to host scarcity or thermal costs of mobility.

Existing studies of patterns of abortion in tsetse have two main gaps. First, studies have not distinguished between abortions in the early and late stages of pregnancy, and egg-stage abortions are often undetected as they are difficult to visualise in the substrate (i.e., leaf litter) and are too small to detect without microscopy. Such distinctions are important, however, when testing adaptive hypotheses because any optimal decision about terminating a reproductive event may depend on how much a female has already invested. In tsetse, the majority of larval growth happens in the final stage of pregnancy (Denlinger and Ma, 1974; Tobe and Langley, 1978; Hargrove and Muzari, 2015) so earlier-stage abortions could reflect more of an adaptive strategy, before females have made any significant investment in their offspring. Second, it is not known how abortion affects subsequent reproduction, which requires tracking the reproductive output of individual females. If females strategically abort offspring to reduce resource loss, they may take less time to produce their next offspring (Saunders, 1960). Alternatively, if abortion is an indication of female stress and this carries over to the next reproductive event, females may take longer to produce their next offspring, and these offspring might also weigh less. Predictions concerning the relationship between abortions and offspring sex are more nuanced, but we hypothesize that females might strategically abort male offspring when they lack sufficient resources to produce enough of both sexes—either as they get older, or due to nutritional stress. This is based on our assumption that females might provide higher fitness returns. The assumption arose from three considerations. First, females are larger (as indicated by wing length) at maturity (Hargrove et al., 2019), even though female and male pupae are similar in weight. Second, females are longer lived (Hargrove et al., 2011). Third, insemination rates in the field are high (90%, Hargrove, 2012). Thus, while we do not know the sex of aborted offspring, there could be a potential bias towards producing female offspring immediately after an abortion if mothers that are old or in poor conditions are likely to abort males.

Here, we investigate patterns of age-specific abortion in tsetse using data from a laboratory-reared tsetse colony. First, we measure stage-specific abortions in females of known age and, second, we track the reproductive history of individual females. We ask whether egg-stage abortions increase with age as observed in previous studies for later, larval-stage abortions, and whether they are also higher in young females as found in field studies. We then use individual-level data to address the question of how an abortion affects the size and sex of the next offspring and the period required to produce it. This leads to potential insights on whether patterns of abortion, according to female age or condition, are due to strategic holding back of female resources or a lack of ability to invest in a particular reproductive event.

## 2 Materials and methods

We analysed the causes and consequences of spontaneous abortions using known-age tsetse, *Glossina morsitans morsitans* Westwood, maintained at the Liverpool School of Tropical Medicine (LSTM). As described in Files S1 and S2 by Lord et al. (2021), the LSTM tsetse colony comprises multiple breeding cages, each with 48 females and 12 males. Each week, 12 new cages are made and placed on a tray (each totaling, initially, 576 females and 144 males) and maintained for up to 3 months. Every week, a new tray is created and the oldest discarded, thus each tray contains age-matched females; and reproductive output of each tray is monitored three times weekly. Unless specified otherwise below, flies were fed defibrinated horse blood (TCS Biosciences Ltd) and maintained at 26°C (±2°C), 72 (±4) % humidity and a photoperiod of 12 h.

Below, we describe two studies conducted on flies from the LSTM colony to understand the causes and consequences of age-specific abortions in female tsetse, one which involves a detailed analysis of stage-specific (i.e., egg and larval) abortions of colony flies (“study 1”) and the other, which involves an experiment that manipulated nutrition in individually-housed flies [as described in Lord et al. (2021), here termed “study 2”]. All statistical analyses were conducted in R version 4.2.1 (R Core Team, 2022) using RStudio version 2022.7.1.554 (RStudio Team, 2022).

### 2.1 Study 1: changes in abortions with maternal age and stage of larval development

First, we conducted analyses on changes in abortion by female age and offspring stage at the colony-level. Previous insights on tsetse abortions have focused on larval stages visible without magnification (e.g., Lord et al., 2021). Here, we used a more detailed approach to quantify abortions from the egg stage for females of known age in the LSTM tsetse colony. In total, we measured the reproductive output of 35 trays, across four time periods (22–29 November 2021 [period 1, *n* = 7 trays]; 21 February 2022 to 6 March 2022 [period 2, *n* = 8 trays]; 7–22 March 2022 [period 3, *n* = 10 trays]; and 23 March 2022 to 8 April 2022 [period 4, *n* = 10 trays]).

**Figure 1:**
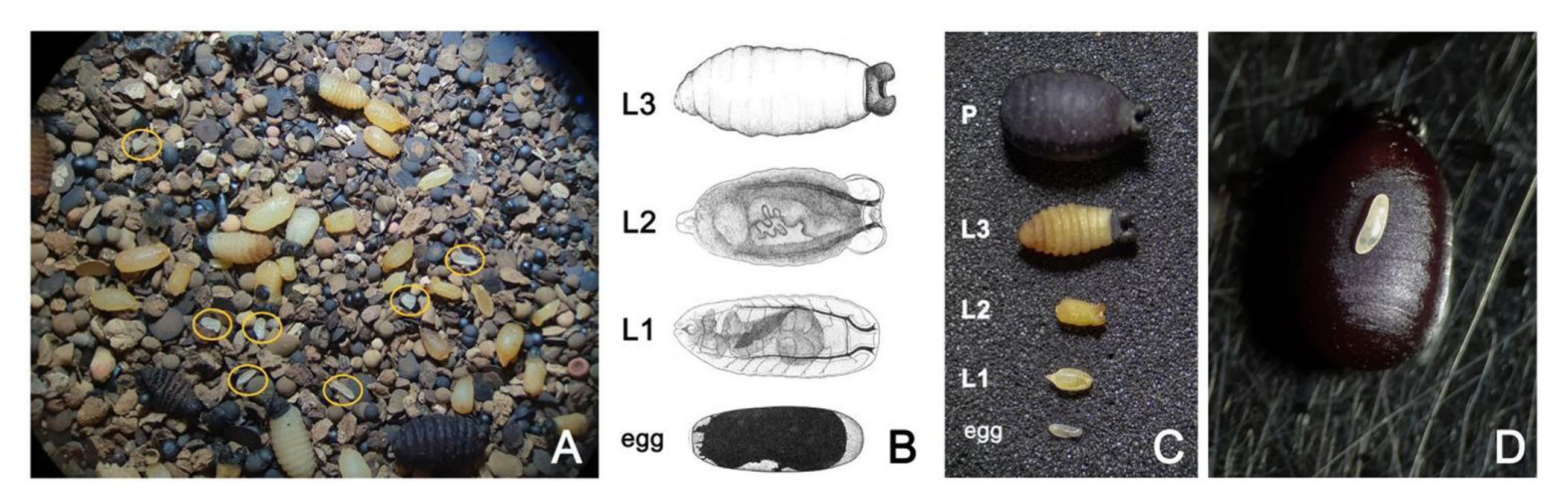
Isolation and characterisation of different stages of tsetse development. **(A)** Petri dish containing material (tsetse dirt) collected from a tray of tsetse aged 3weeks containing fly frass, and premature larval and egg (seven yellow circles) depositions. **(B)** Detailed diagrams of each gestational stage [modified from Burtt and Jackson (1951)]: from egg to mature 3rd instar larva. **(C)** The reproductive losses collected from each tray were scored as: egg, L1: first instar larva lacking polypneustic lobes, L2: second instar larva with distinct polypneustic lobes, L3: third instar larva with distinct body segmentation and black polypneustic lobes, P: full term pupated larva (pupa). **(D)** Size comparison between an egg and the extraordinary exponential growth it undergoes in 4–5days to become a pupa.

During each period, we checked the colony trays three times per week to record viable births (numbers of pupae deposited and total pupal mass), as well as abortions. For the latter, we collected material from below the colony cages and, using a dissecting microscope (40X), examined the “tsetse dirt” (Figure 1A), which is a collection of frass, spermatophores, aborted eggs and larvae to detect aborted offspring of various developmental stages. These were categorised as: (1) Egg: smooth, intact pearlescent egg with a defined pellicle, (2) L1: larva is small, opaque and pale yellow, with no visible polypneustic lobes, (3) L2: larva is opaque, darker yellow, polypneustic lobes are formed but not melanised, and (4) L3: black polypneustic lobes are visible and larval segmentation is evident (Figures 1B–D). Counts were made per-tray. For periods 1 and 2, the maximum ages of females recorded were 9 and 10 weeks respectively, whereas for periods 3 and 4 abortion data were available for females up to the age of 12 weeks. Note that <10% of females die before the age of 12 weeks (Haines et al., unpublished data).

We analysed how stage-specific abortions vary across maternal age by fitting a generalised additive model to the data to capture any non-linear patterns, using the R package “mgcv” (Wood, 2017). We fit GAMs with knots (i.e., points at which the slope changes) varying from 3 to 10 and compared models using Akaike Information Criteria, corrected for small sample sizes (AICc). Plots were made using the GAM for each stage with the lowest AICc.

### 2.2 Study 2: (i) estimating presumed abortions in individually tracked females and (ii) quantifying effect of known or presumed previous abortion on subsequent larviposition duration, offspring weight and offspring sex

We conducted an experiment on individually-caged females, originating from the same colony, to establish the effect of nutritional stress and mating history on age-dependent reproductive allocation [see detailed methods in Lord et al. (2021)]. Previously, we considered the overall effects of reduced nutrition and delayed mating on offspring quality and survival across female age. Here, we investigate individual-level consequences of abortion for subsequent reproduction; and any potential bias towards producing a particular sex in older females or following an abortion. We focus on the contrast between nutritionally stressed females and those fed the rearing standard defibrinated horse blood (“control”). Nutritionally stressed females were provided a bloodmeal adjusted with serum to have a lower packed cell volume (hematocrit, PCV)—10% PCV versus a control of 45% PCV—to mimic an animal sick with anaemia-related trypanosomiasis (Mamoudou et al., 2015).

Previous studies on laboratory and field tsetse have found that the inter-larval period follows a consistent timing for flies kept at constant temperature, being, on average, 16 days to produce the first larva and 9 days for subsequent larvae at 25°C (Mellanby, 1937; Denlinger and Ma, 1974; Hargrove, 2004). However, when we tracked reproductive output of individual females (*n* = 190) mated within 48 h of emergence, the inter-larval period was often considerably longer than expected—even when known larval abortions were excluded, being up to 52 days to produce the first larva and up to 32 days between subsequent larvae. We postulated that such variability may be due to the presence of egg-stage, or very early larval-stage, abortions which were not recorded in this experiment. Henceforth, we applied the Hampel filter (Pearson, 2002) to our dataset to establish an unbiased threshold for detecting such assumed egg abortions. This works by deeming as outliers any data points 3 or more median absolute deviations (MAD) from the median.

We analysed whether such presumed abortions are higher in nutritionally stressed females, and increase with female age, in line with our previous findings regarding confirmed abortions (Lord et al., 2021). For this analysis, we excluded known abortions, and coded each gestation interval as having produced offspring within a normal interval (0), or having taken too long (1, hence likely including an undetected abortion based on the threshold as described above). We then conducted a binomial GLM, on *n*=93 control flies and *n*=86 nutritionally stressed flies, including as covariates experimental treatment, and linear and quadratic maternal age effects. Maternal age was *z*-transformed. We established the significance of input variables by inspecting the effect size and standard error for each term included in the main model.

We were also interested in whether females that aborted an offspring could then accelerate the time to produce their next larva, and what the consequences of a previous abortion were for the size and sex of subsequent offspring. We thus compared the larviposition duration, pupal wet weight (in mg) and offspring sex after a known viable birth to those produced after a recorded abortion, or after a presumed abortion. For this analysis, we included *n* = 91 control flies and *n* = 77 nutritionally stressed flies.

We analysed the effect of previous birth outcome on subsequent birth outcome using linear mixed-effect models (LMM), for larviposition duration and offspring weight, and general linear mixed-effect models (GLMM), for offspring sex, using the R library lme4 (Bates et al., 2015). We considered only those larvipositions where a viable pupa was produced, within the accepted thresholds for a larviposition (i.e., 8–14 days). For each model, we included previous birth outcome (no abortion, confirmed abortion or presumed abortion in previous inter-larval period) as an input variable, and accounted for maternal treatment (nutritional stress or control) and *z*-transformed maternal age (linear and quadratic) as potential covariates. We also included interactions between previous birth outcome and treatment to ask whether any carryover effects from previous larvipositions depend on maternal nutritional stress. Maternal identity was included as a random effect to account for repeated measures on individual mothers. As above, significance of input variables was established by inspecting the effect size and standard error for each term included in the main model, and by conducting ANOVA comparisons of models.

## 3 Results

### 3.1 Study 1: changes in abortions with maternal age and stage of larval development

**Figure 2:**
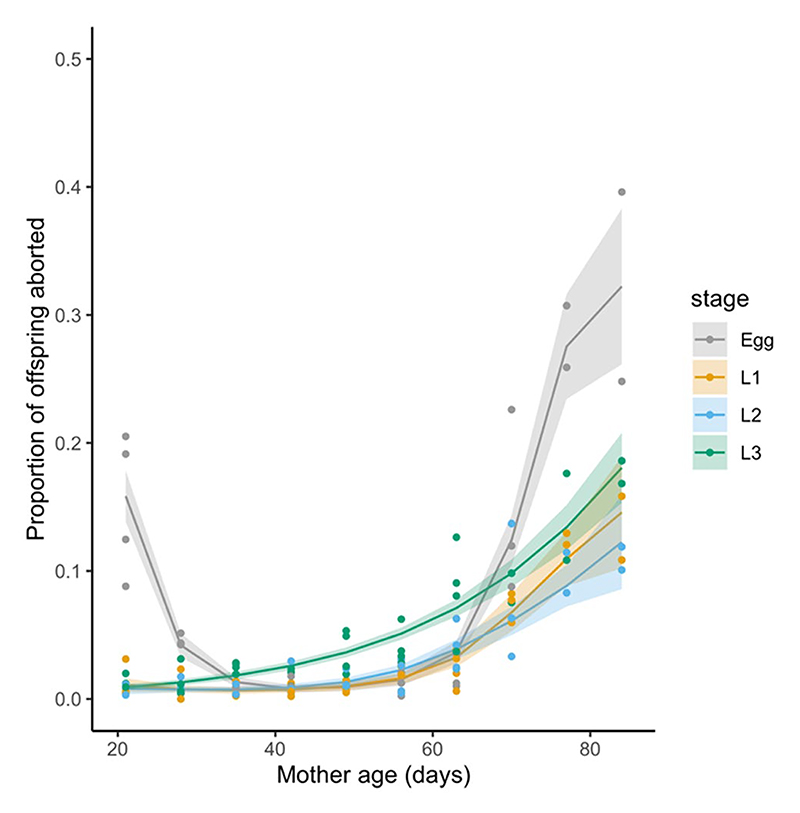
Abortions, as a proportion of total pregnancies (i.e., pupae plus abortions) and as a function of female age in days. Different colours indicate the different developmental stages considered (egg, L1, L2, or L3 larvae). Individual points represent data for each collection period, lines and shading indicate predictions and 2×standard error from a GAM fitted to each developmental stage.

When we consider stage-specific abortions, we find that egg abortions are relatively more frequent in younger females (when larval abortions are rare) and again in older flies (Figure 2), whereas larval-stage abortions (L1, L2, or L3) are rare in younger females and increase in frequency as females get older. Overall, there was a trend for there to be higher egg-stage abortions than abortions at later larval stages (Figure 2)—particularly for very young and older females—however, due to low sample sizes, formal statistical comparison was not possible.

### 3.2 Study 2: (i) estimating presumed abortions in individually tracked females and (ii) quantifying effect of known or presumed previous abortion on subsequent larviposition duration, offspring weight and offspring sex

Applying the Hampel filter to our experimental data, we established 16−28 days as an interval for females to produce their first larva without any undetected abortion; and 8−14 days between subsequent larvae. We accept that these intervals are wider than reported inter-larval periods at constant temperature, but we use these thresholds as a more conservative and unbiased approach based on data distributions.

**Figure 3:**
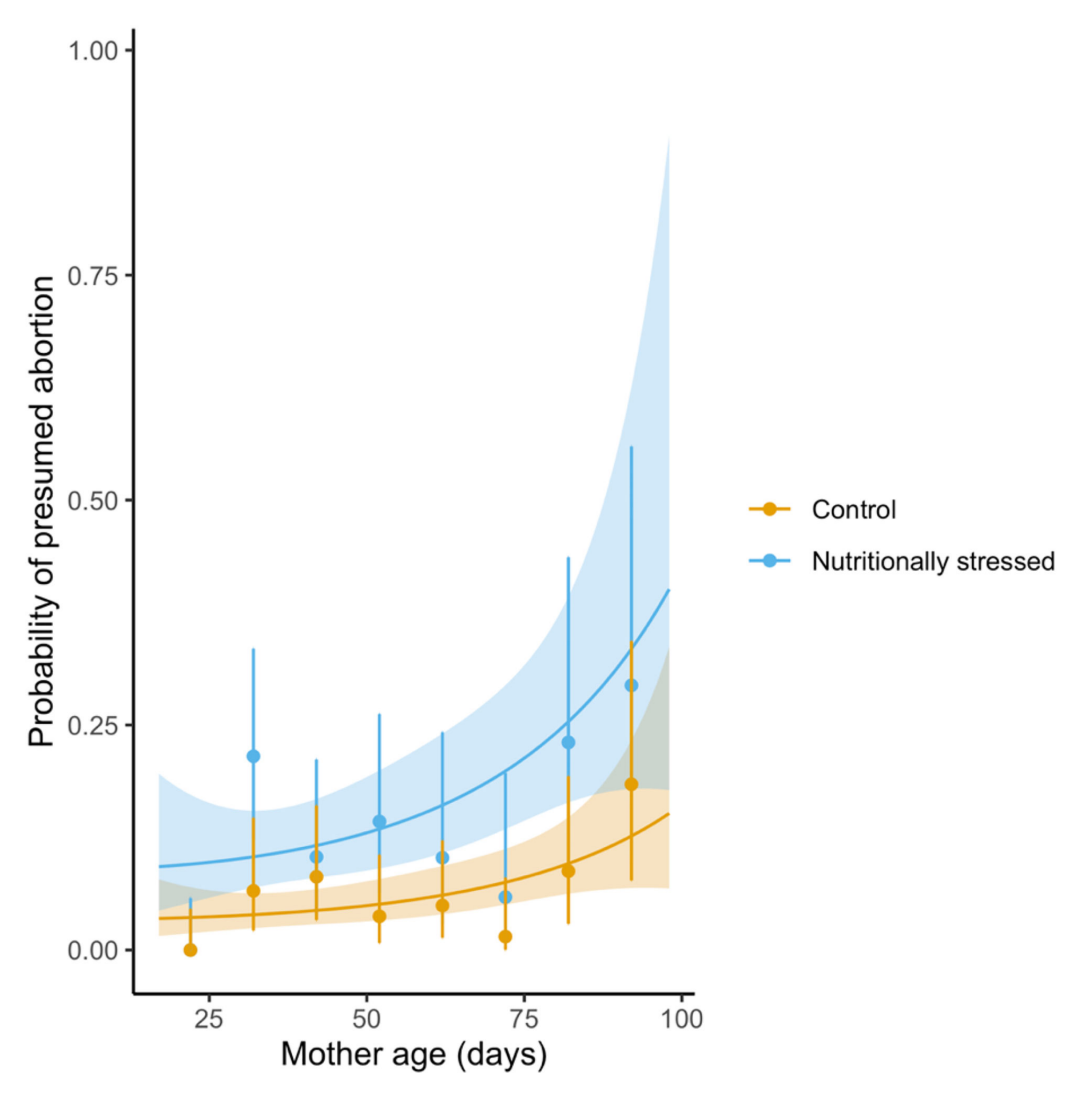
Probability of a presumed abortion per inter-larval period—based on being longer than the expected, typical period—as a function of maternal diet (blue=nutritionally stressed, 10% PCV blood treatment, orange=control 45% PCV blood treatment) and age. Lines denote predicted probabilities based on a GLMM fit to the data, with 95% prediction intervals (shaded area); and points are the means for 10-day periods with exact binomial 95% confidence intervals.

We found that, as shown in our previous work on confirmed larval abortions, the incidence of presumed egg or larval abortions— based on the inter-larval period being above the threshold of 28 days (first larva) or 14 days (subsequent larvae)—is higher in females who experienced nutritional stress (effect of treatment: 0.971 ± 0.246, *z* = 3.94, *p* < 0.0001, Figure 3). We did not, however, find any significant association in the likelihood of a presumed abortion and maternal age, with both linear and quadratic terms of maternal age having small effect sizes (0.00052 and 0.00016, *p* = 0.986 and 0.23 respectively).

**Figure 4:**
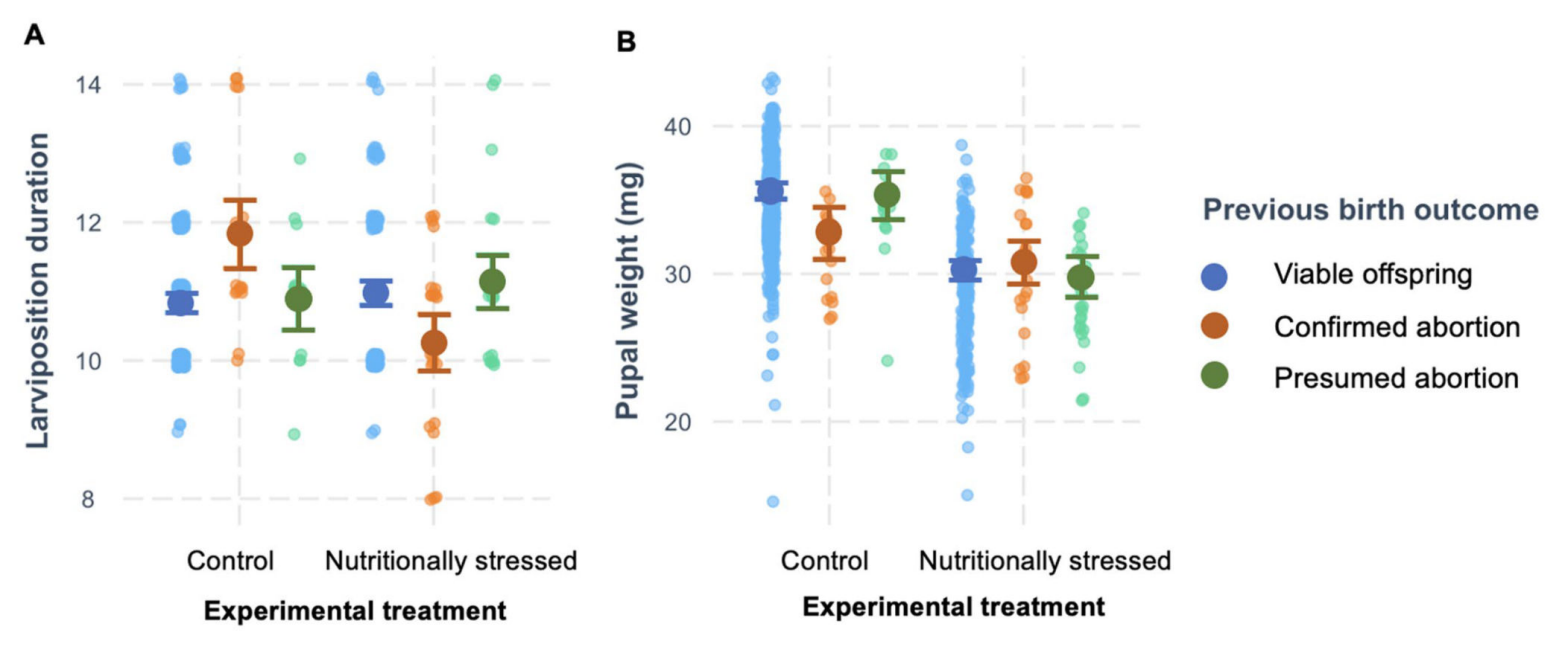
Effect of previous birth outcome on **(A)** subsequent larviposition duration and **(B)** subsequent pupal weight. Shown are the raw data (points), and predictions with 95% prediction intervals (generated using the cat_plot function in R library “interactions”, Long, 2019) from the LMM on larviposition duration and pupal weight, respectively, depending on maternal treatment (control or nutritionally stressed).

We considered whether having an abortion (either confirmed or presumed) affected the time taken to produce the next viable offspring, its mass, or its sex (Table 1). We found that experiencing an abortion did influence time taken to produce the next offspring, although this depended on maternal experimental treatment (effect of previous outcome × treatment: F_2,690_ = 14.33, *p* < 0.0001, Figure 4A). Control females, raised on a diet of undiluted blood, took on average 1.02 days longer to produce their next offspring after aborting a previous one (when such an abortion was observed rather than presumed) compared to when they produced a viable offspring. In contrast, nutritionally stressed females, fed diluted blood, took slightly less time (0.68 days) to produce their next offspring after a confirmed abortion. Maternal age (as linear or quadratic term) did not affect the time taken to produce the next viable offspring (Table 1).

When we considered the effect of previous birth outcome on subsequent offspring mass, we found, again, that any effect depended on maternal experimental treatment (effect of previous outcome × treatment: F_2,666_ = 4.54, *p* = 0.011, Table 2; Figure 4B). Specifically, females on the control diet produced smaller offspring after having an observed abortion than after a normal birth or presumed abortion; whereas there was no difference among nutritionally stressed females in the size of offspring produced depending on previous birth outcome. As shown previously, we found both the linear and quadratic term for maternal age to be significant in the model (Table 2).

We found no effect of previous birth outcome on whether the next offspring produced was a male or female (χ^2^ = 2.74, *p* = 0.254), nor was the probability of a sex bias affected by maternal age or experimental treatment (Table 3).

## 4 Discussion

We used detailed observations on stage-specific abortions, and experimental data tracking individual reproductive output, to investigate age-dependent patterns of early-reproductive failure in laboratory tsetse. We found that, in contrast to previous work—where larval abortions increase with age in females—egg-stage abortions are also elevated in younger females. Taken together, these abortion patterns follow a similar U-shaped (or inverse-bell-shaped) curve consistent with studies on foetal loss in humans and baboons, and which would fit both adaptive- and constraint-based predictions about age-dependent changes in reproductive loss. We then analysed individual data to investigate the consequences of experiencing an abortion for subsequent reproductive outcomes. We found that there is no clear evidence that abortion is an adaptive strategy to improve future reproductive success; after an abortion, females do not generally take less time to produce their next offspring, these offspring are not larger, and they do not produce more females (the sex with higher presumed fitness returns, living longer and being more likely to produce offspring consistently when mated).

Our study is the first to quantify, using detailed observation of the debris below tsetse breeding cages, how egg-stage abortions change across female age. Previous studies have focused on larval-stage abortions—which are observable without inspection under a microscope—and have shown that, in the laboratory, such abortions increase as females get older (Lord et al., 2021). In general, we find a relatively high number of egg-stage abortions compared to later stage abortions, which indicates that these are likely under-represented in other studies. We also find that egg-stage abortions increase as females get older, but these are also relatively high in younger females. Thus, the U-shaped pattern of abortion fits with our other work—and more general observations of age-dependent reproductive output (Monaghan et al., 2020)—that both younger and older females have compromised reproduction (smaller offspring, higher pre-natal mortality). Higher rates of reproductive loss in younger females also align with field studies in tsetse, which show that younger females tend to produce smaller offspring (English et al., 2016; Hargrove et al., 2018) and have a higher proportion of empty uteri, which is indicative of a recent abortion (Hargrove, 1999). Females producing their first offspring may not be in physiologically prime condition (Haines, 2013); early in the first pregnancy they may have lower levels of fat thoracic musculature than fully mature flies and so have less aptitude at host searching (Hargrove, 1975). Moreover, they may have lower fat stores and thus be unable to provide sufficient milk for a successful pregnancy. Given that they are at the onset of their reproductive career, terminating reproductive events early in gestation may be an adaptive strategy to retain resources for their own maintenance and future reproduction. We are not aware of any theoretical models on maternal allocation with offspring prenatal mortality across different developmental stages as an outcome: future work investigating different adaptive strategies of withholding allocation early or late in pregnancy depending on female age and physiological condition will yield predictions for empirical studies.

Our analyses both on confirmed and presumed pregnancy losses (based on longer than typical inter-larval period) highlight the strong effect of female nutritional stress on elevated abortion rates across all ages in tsetse. Such a link between stress and abortion has been shown previously in tsetse, when considering stress in terms of nutritional deprivation (Mellanby, 1937; Saunders, 1972; Lord et al., 2021), exposure to insecticides or toxicants in bloodmeals (Saunders, 1971; Riordan, 1986) and, in field studies, exposure to heat stress (Hargrove, 1999). These findings resonate with a recent study on wild hybrid baboons which showed that foetal loss is higher when females experience high temperatures late in pregnancy, and when they occupy a poor-quality habitat (Fogel et al., 2023). There is increasing appreciation that climate change and extreme heat will affect not just survival but fertility across a range of species (Walsh et al., 2019), including humans, but owing to difficulty in measuring prenatal loss or reproductive failure, fewer studies focus on this as an outcome. Modelling of tsetse populations tends to focus on adult mortality and pupal emergence rates, which are impacted by host availability and increasing maximum temperatures (Lord et al., 2018). Findings from this study, and related field studies, highlight how such environmental stress also affects prenatal mortality and should thus be considered in future modeling work to predict tsetse population responses to climate change and the emergent disease dynamics.

We acknowledge that our findings are limited to the laboratory context and caution should thus be applied when generalising to field conditions. In the laboratory, tsetse mortality rates are lower as there is no predation and flies are offered regular and predictable access to blood. Laboratory females do not have the opportunity to fly around freely, and they thus accumulate fat as they get older, and this may compromise reproductive output. This could help explain why older females in the laboratory, but not the field, have higher abortion rates and produce smaller offspring (Lord et al., 2021). Importantly, when considering whether a pregnant female carries a larva to term, the timing of bloodmeals is key: in the field, females can choose the optimal time to feed depending on the size of their larva, whereas laboratory females may be offered blood when at a suboptimal stage of gestation. Lack of flexibility of feeding may thus explain higher abortion rates in the laboratory (Mellanby, 1937). The microbial community—which plays an important role in tsetse reproduction (Nogge, 1976; Alam et al., 2011)—of field versus laboratory flies is also different, which makes it hard to compare them directly. In general, findings in laboratory studies such as this give insights into the physiological processes and capacity of females, but parallel work on field-caught flies—where possible (see below for limitations)—would complement this work and validate the ecological relevance.

At the same time, it is important to appreciate the unique insights to be gained from such detailed laboratory studies, which would not be possible in a field setting. For example, the lack of data on egg abortions in tsetse has largely been due to the difficulty of observing reproductive output at this stage—which requires inspecting all tsetse-related debris from a breeding cage under a microscope. Such observations would not be possible in the field. Similarly, we were interested in investigating the consequences of abortion for subsequent reproduction, which requires longitudinally tracking the reproductive outputs of individual females. Such within-individual data are not available in field studies, which limits inference as we cannot tease apart between-individual effects (e.g., better condition flies living longer) versus within-individual effects (e.g., previous birth outcome affects subsequent reproduction). When we considered such individual-level data, we find that—for females fed control (undiluted) blood—after having an abortion, they take longer to produce their next offspring, and these offspring are smaller. This suggests that abortion may be more likely due to physiological constraint than an adaptive strategy to improve subsequent reproductive output. This suggestion is tentative, however, given the weak effect and small sample size, and future work on this topic will be enlightening. Moreover, we do not find that the sex of viable offspring is affected by the age, nutritional state or reproductive history of the mother. We had predicted that females might strategically abort male offspring when they lack sufficient resources to produce a balanced sex ratio; but we note that this is built on our assumption that female tsetse are the sex with higher fitness returns. There is a need for more research to test this assumption and to develop theoretical predictions on whether sex biases may occur in tsetse depending on mode of sex determination, energetic requirements of males or females as well as their likely reproductive success.

Our study focuses on abortions (termed also prenatal mortality, reproductive failures or foetal/larval loss) in a viviparous fly. We provide new evidence showing that, when considering the developmental stage of offspring aborted, younger females are more prone to abort eggs and abortions increase overall with female age, as reported previously. We do not find strong evidence, however, that abortion is an adaptive strategy to improve subsequent reproductive output. As found in field studies, abortion rates are indicative of female physiological stress—here measured as nutritional deprivation. We appreciate the challenges of measuring such prenatal mortality as a reproductive outcome in the field (both in tsetse and other viviparous taxa). Such challenges not withstanding, we argue that this represents a key reproductive trait with important implications both for understanding age-dependent maternal allocation as well as how ecological adversity, and climate change, will impact population dynamics in these species.

## Figures and Tables

**Table 1:** Full model results for linear mixed model (LMM) on time taken to produce next viable offspring depending on previous birth outcome.

	Estimate	SE	df	*t* value	*P*-value
Intercept	10.83	0.07	218.84	155.20	<0.0001
Previous abortion (confirmed)	1.00	0.25	690.60	4.04	<0.0001
Previous abortion (presumed)	0.07	0.23	690.48	0.28	0.779
Experimental treatment (nutritional stress)	0.15	0.10	190.41	1.46	0.147
Maternal age (𝓏-transformed)	0.01	0.04	632.41	0.24	0.814
Maternal age^2^ (𝓏-transformed)	−0.01	0.03	608.53	−0.33	0.742
Previous outcome (confirmed) × Treatment (nutrition)	−1.72	0.32	690.92	−5.31	<0.0001
Previous outcome (presumed) × Treatment (nutrition)	0.10	0.31	689.23	0.32	0.752

Model based on *n* = 699 observations of 168 females (random effect variance ± SD: 0.165 ± 0.406).

**Table 2:** Full model results for linear mixed model (LMM) on weight of next viable offspring depending on previous birth outcome.

	Estimate	SE	df	*t* value	*P*-value
Intercept	35.66	0.27	188.41	130.38	<0.0001
Previous abortion (confirmed)	−2.87	0.88	669.21	−3.27	0.001
Previous abortion (presumed)	−0.33	0.83	670.98	−0.40	0.688
Experimental treatment (nutritional stress)	−5.40	0.40	176.59	−13.40	<0.0001
Maternal age (𝓏-transformed)	0.97	0.12	593.70	7.80	<0.0001
Maternal age^2^ (𝓏-transformed)	−1.16	0.12	562.96	−10.00	<0.0001
Previous outcome (confirmed) × Treatment (nutrition)	3.41	1.14	667.90	3.00	0.003
Previous outcome (presumed) × Treatment (nutrition)	−0.10	1.07	665.41	−0.09	0.927

Model based on *n* = 682 observations of 168 females (random effect variance ± SD: 3.702 ± 1.924).

**Table 3:** Full model results for generalised linear mixed model (GLMM) on sex of next viable offspring depending on previous birth outcome.

	Estimate	SE	*z* value	*P*-value
Intercept	0.02	0.17	0.11	0.915
Previous abortion (confirmed)	0.21	0.66	0.32	0.747
Previous abortion (presumed)	−0.57	0.77	−0.73	0.464
Experimental treatment (nutritional stress)	0.00	0.25	0.02	0.985
Maternal age (𝓏-transformed)	−0.21	0.11	−1.87	0.061
Maternal age^2^ (𝓏-transformed)	0.09	0.10	0.92	0.36
Previous outcome (confirmed) × Treatment (nutrition)	0.04	0.91	0.04	0.969
Previous outcome (presumed) × Treatment (nutrition)	−0.27	0.98	−0.28	0.779

Model based on *n* = 413 observations of 156 females (random effect variance ± SD: 0.148 ± 0.385).

## Data Availability

The datasets presented in this study can be found in online repositories. The names of the repository/repositories and accession number(s) can be found below: GitHub, https://github.com/sineadenglish/tsetse-repro-loss.
